# Effects of Cricket Powder Supplementation on Gut Microbiota in High-Fat Diet-Fed Mice

**DOI:** 10.3390/foods15081328

**Published:** 2026-04-10

**Authors:** Yanjun Guo, Rui-Qi Shi, Ananya Dechakhamphu, Min Zhao, Ju-Sheng Zheng, Sirithon Siriamornpun

**Affiliations:** 1 Department of Food Technology and Nutrition, Faculty of Technology, Mahasarakham University, Kantarawichai 44150, Maha Sarakham, Thailand; 67010862501@msu.ac.th; 2 Research Center for Industries of the Future, School of Medicine, School of Life Sciences, Westlake University, Hangzhou 310024, China; shiruiqi@westlake.edu.cn; 3 Thai Traditional Medicine Program, Faculty of Thai Traditional and Alternative Medicine, Ubon Ratchathani Rajabhat University, Ubonratchathani 34000, Thailand; ananya.d@ubru.ac.th; 4 Cosmetic Science and Spa Program, Faculty of Thai Traditional and Alternative Medicine, Ubon Ratchathani Rajabhat University, Ubonratchathani 34000, Thailand; 5 Yunnan Key Laboratory of Breeding and Utilization of Resource Insects, Institute of Highland Forest Science, Chinese Academy of Forestry, Kunming 650224, China; mzhao@caf.ac.cn; 6 School of Medicine, Westlake University, Hangzhou 310024, China; zhengjusheng@westlake.edu.cn; 7 Research Unit of Thai Food Innovation, Department of Food Technology and Nutrition, Mahasarakham University, Kantarawichai 44150, Maha Sarakham, Thailand

**Keywords:** Microbiome, dyslipidemia, mouse model, metabolic parameters, 16S rRNA sequencing

## Abstract

The gut microbiota plays an important role in host physiology and is highly influenced by dietary factors. This study aimed to investigate the effects of cricket powder (CP) supplementation on gut microbiota composition in high-fat diet-fed C57BL/6J mice. Male C57BL/6J mice were fed a normal diet or a high-fat diet. Mice fed the high-fat diet were administered low, medium, or high doses of CP by gavage. Serum lipid levels and liver-related biochemical indicators were measured, and gut microbiota composition was analyzed using 16S rRNA gene sequencing. We found that CP supplementation significantly (*p* < 0.05) altered gut microbiota diversity and community structure, with differences observed among CP doses. Alpha diversity indices were significantly reduced after the intervention (*p* < 0.05). Beta diversity analysis showed no significant separation among groups before the intervention, whereas a clear separation in gut microbiota structure was observed after the intervention. Correlation analysis further revealed that beneficial bacterial genera, including *Lactobacillus*, *Bifidobacterium*, and *Akkermansia*, were negatively associated with lipid-related parameters. Overall, these findings suggest that CP supplementation can modulate gut microbiota composition under high-fat dietary conditions, indicating its potential role in metabolic regulation.

## 1. Introduction

Cardiovascular disease remains the leading cause of death and disability worldwide, accounting for an estimated 19.8 million deaths in 2022 (WHO). As a major and modifiable risk factor for cardiovascular diseases, hyperlipidemia plays a critical role in disease prevention and intervention [1]. Hyperlipidemia refers to a group of lipid metabolism disorders characterized by abnormally high levels of harmful lipids in the blood. Its main manifestations include elevated serum total cholesterol (TC), triglycerides (TG), and low-density lipoprotein cholesterol (LDL-C), along with decreased high-density lipoprotein cholesterol (HDL-C) levels [2]. Recent epidemiological data indicate that the prevalence of dyslipidemia among adults is approximately 37.5% in China and 40.9% in South Korea, while recent studies from Thailand report that about 66.5% of the population exhibits varying degrees of dyslipidemia [3,4,5]. This metabolic disorder not only increases the risk of cardiovascular diseases such as atherosclerosis, coronary heart disease, and stroke, but is also closely related to the development of various metabolic-related diseases such as obesity and diabetes [6]. Although statins are widely used as first-line therapeutics for the clinical management of hyperlipidemia, their application is to some extent limited by adverse effects, and therefore, the development of safe, natural, and effective health food supplements has become a current research hotspot [7].

With the continuous growth of the global population and increasing constraints on arable land, identifying food sources that are both sustainable and nutritionally adequate has become a major challenge [8]. In this context, edible insects have attracted considerable attention as alternative protein sources due to their high nutritional value and lower environmental impact compared with conventional livestock [9]. Among them, crickets are one of the most widely studied and utilized edible insects [10]. Cricket powder (CP) is a natural food raw material with high protein and rich nutrition. Its dry weight protein content is about 60–70%, and it is also a high-quality source of essential amino acids, which play an important role in maintaining normal physiological functions. In addition, CP contains polyunsaturated fatty acids, which are considered beneficial for cardiovascular health, as well as various vitamins and minerals such as zinc and potassium [11,12]. Beyond its nutritional value, CP also contains functional components, including chitin and chitosan, which may exert health-promoting effects by modulating the gut microbiota. These components have been reported to promote the proliferation of beneficial bacteria such as *Lactobacillus* and *Bifidobacterium* [13,14]. Such modulation of gut microbiota may subsequently influence host metabolic parameters, particularly lipid metabolism, thereby contributing to the prevention of cardiovascular diseases.

Increasing evidence suggests that gut microbiota dysbiosis is closely associated with the development of metabolic disorders, including obesity, diabetes, and dyslipidemia [15]. Alterations in gut microbial composition may trigger chronic low-grade inflammation and disrupt normal lipid metabolism [16]. Dietary factors are among the major external drivers shaping gut microbiota structure. Insufficient n-3 PUFA intake may shift the gut microbiota toward a higher Firmicutes-to-Bacteroidetes ratio, a feature commonly associated with metabolic dysfunction [17]. However, the specific effects of cricket powder on gut microbiota composition, particularly under hyperlipidemic conditions, remain unclear.

Based on this background, the present study employed a high-fat diet-induced mouse model to systematically evaluate the effects of CP supplementation on gut microbiota composition and diversity. In addition, the potential associations between CP supplementation, gut microbiota modulation, and lipid metabolism-related parameters were further explored, with the aim of providing experimental evidence for the application of CP as a dietary strategy to improve metabolic health and reduce the risk of cardiovascular diseases.

## 2. Materials and Methods

### 2.1. Preparation of Cricket Powder

The preparation method and nutritional composition of the CP were based on our laboratory’s previous research [18]. The CP was obtained from a local farm in Thailand. Crickets were harvested at the adult stage and immediately stored frozen. Following the method of Udomsil et al. [19], with slight modifications, the samples were thawed, boiled, and then dried at 60 °C for 8 h before being ground into fine powder and passed through a 60-mesh (250 µm) sieve to ensure a uniform particle size for further use.

### 2.2. Animals

A total of 30 male C57BL/6J mice (7 weeks old, 20 ± 2 g) were obtained from Zhejiang Vital River Laboratory Animal Technology Co., Ltd. (Jiaxing, Zhejiang, China; license number: SCXK (Zhe) 2024-0001). They were housed at 20–24 °C under a 12 h light/12 h dark cycle, with free access to standard pellet diet and distilled water. Animals were acclimated for 1 week before the start of the experiments and were fed a standard chow diet during the acclimatization period. The study was approved by the Ethics Committee of Westlake University (No. AP: 25-175-JU-SHENG ZHENG).

### 2.3. Experimental Procedures

**Figure 1:**
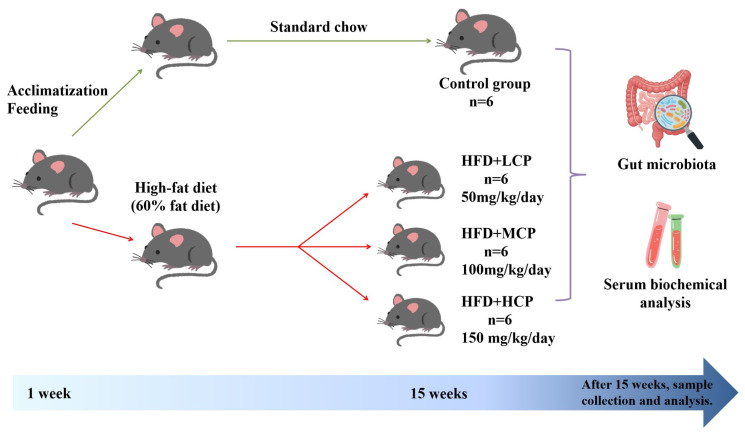
Experimental designs and procedures.

After one week of acclimatization, the mice were randomly divided into five groups: a control group (CON, fed a standard diet), a high-fat diet group (HFD, fed a 60% fat diet), and three CP intervention groups receiving low (HFD+LCP, 50 mg/kg/day), medium (HFD+MCP, 100 mg/kg/day) and high (HFD+HCP, 150 mg/kg/day) doses, respectively [20]. To ensure similar mean body weight across groups at baseline, animals with comparable weights were distributed among groups. Investigators were not blinded to group allocation during treatment and data analysis. Before gavage, CP was dissolved in 0.5% sodium carboxymethyl cellulose (CMC-Na) solution, while the control and model groups received an equal volume of CMC-Na [21]. The intervention was administered by oral gavage for 15 consecutive weeks. During the experimental period, all mice had free access to food and water, and body weight was recorded weekly. On the final day of the experiment, the mice were fasted for 12 h prior to anesthesia, and blood samples were collected from the eyeballs, as shown in Figure 1.

### 2.4. Oral Glucose Tolerance Test (OGTT)

An oral glucose tolerance test (OGTT) was performed at the end of the experimental period. After 12 h of fasting, mice were orally administered glucose (2.0 g/kg body weight). Blood glucose levels were measured from the tail vein at 0, 15, 30, 60, and 120 min using a glucometer. The area under the curve (AUC) was calculated to evaluate glucose tolerance.

### 2.5. Serum Biochemical Analysis

Serum levels of triglycerides (TG), total cholesterol (TC), low-density lipoprotein cholesterol (LDL-C), high-density lipoprotein cholesterol (HDL-C), aspartate aminotransferase (AST), and alanine aminotransferase (ALT) were measured using an automated biochemical analyzer (ProCyte Dx, Westbrook, ME, USA).

### 2.6. Gut Microbiota Analysis

Fecal samples from mice were collected and stored at −80 °C until analysis. Genomic DNA was extracted from the feces of each group using the FastPure Stool DNA Isolation Kit (MJYH, Shanghai, China). DNA quality and integrity were assessed by 1% agarose gel electrophoresis. The V3–V4 region of the bacterial 16S rRNA gene was amplified using the universal primers 338 F (5′-ACTCCTACGGGAGGCAGCAG-3′) and 806 R (5′-GGACTACHVGGGTWTCTAAT-3′). Sequencing was performed on the Illumina Nextseq 2000 platform by Shanghai Majorbio Bio-pharma Technology Co., Ltd. (Shanghai, China).

Raw sequences obtained from 16S rRNA gene sequencing were quality-filtered and processed using QIIME 2 to construct feature tables. Taxonomic assignment was performed based on the SILVA reference database. Alpha diversity indices, including the Shannon index, evenness, and observed features, were calculated using QIIME 2 and subsequently exported for statistical analysis and visualization using GraphPad Prism8.0.2. For beta diversity analysis, Bray–Curtis dissimilarity matrices were calculated based on the feature data, and non-metric multidimensional scaling (NMDS) was performed using R software (version 4.5.2) to visualize differences in gut microbiota community structure among samples. Statistical analysis and visualization of the annotated feature data, including relative abundances of gut microbiota at the phylum and genus levels, were also conducted using R software.

### 2.7. Statistical Analysis

Results are expressed as mean ± standard error of the mean (SEM). Statistical analyses were performed using one-way analysis of variance (ANOVA), followed by Tukey’s multiple comparisons test, using GraphPad Prism version 8.0.2. Differences were considered statistically significant at *p* < 0.05.

## 3. Results

### 3.1. Body Weight and Blood Glucose Profiles

**Figure 2:**
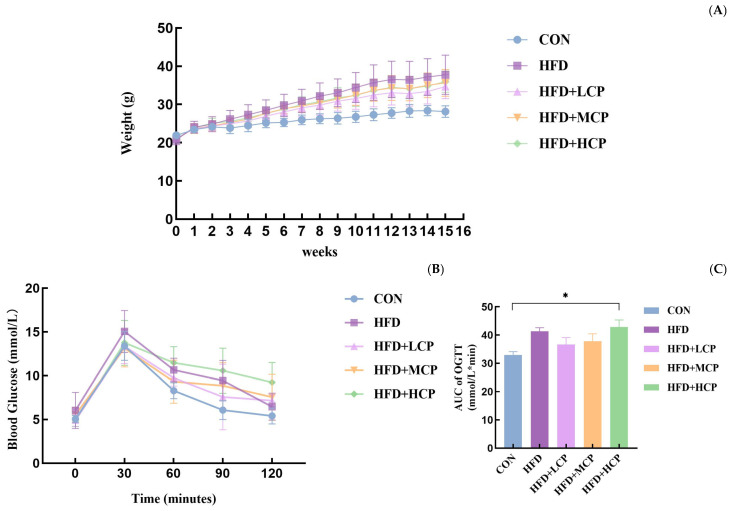
Body weight and glucose tolerance in mice. (**A**) Changes in body weight during the experimental period. (**B**) Blood glucose levels measured during the oral glucose tolerance test (OGTT) at the end of the experimental period. (**C**) Area under the curve (AUC) of the OGTT. Data are presented as mean ± SEM. Statistical analysis was performed using one-way ANOVA. *p* < 0.05 was considered statistically significant. * *p* < 0.05 compared with the CON group.

Body weight is shown in Figure 2A. During the 15-week intervention, the HFD group exhibited a significantly higher body weight compared with the control group.

In the oral glucose tolerance test, all groups showed similar glucose curves with a peak at 30 min (Figure 2B). No statistically significant differences were observed among the groups. Although the HFD+LCP and HFD+MCP groups tended to have lower glucose levels and lower area under the curve (AUC) values compared with the HFD group, these differences did not reach statistical significance (Figure 2C).

### 3.2. Effects on Serum Lipids and Liver Enzymes

**Figure 3:**
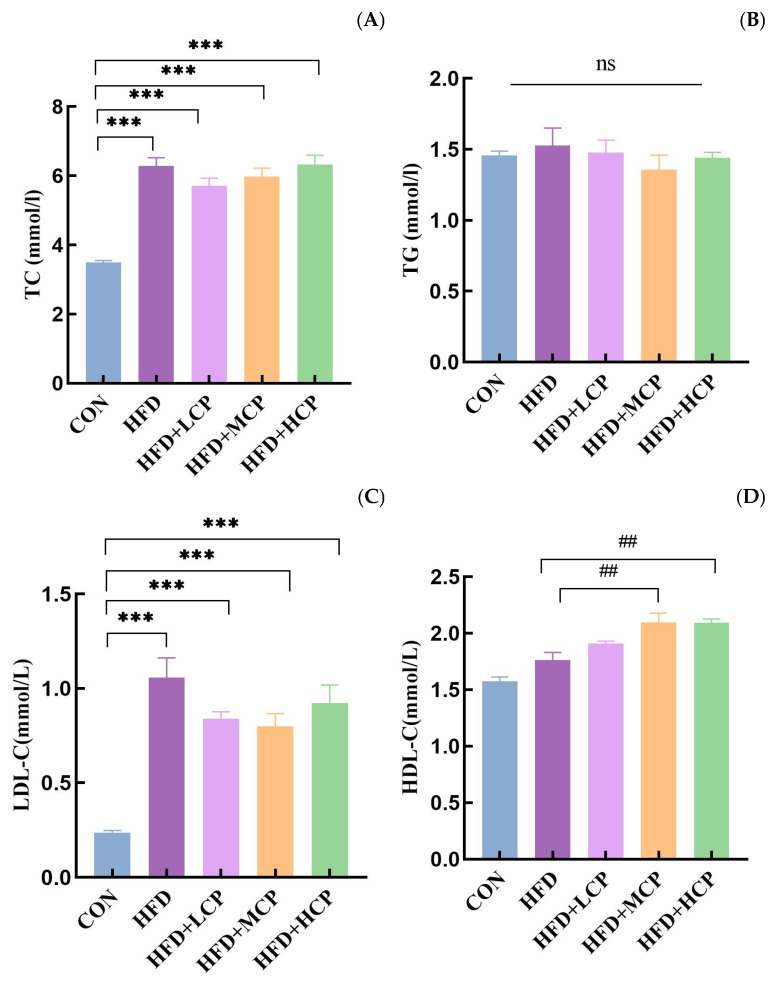
Effects of CP intervention on serum lipid profiles. (**A**) Serum total cholesterol (TC) levels. (**B**) Serum triglyceride (TG) levels. (**C**) Serum low-density lipoprotein cholesterol (LDL-C) levels. (**D**) Serum high-density lipoprotein cholesterol (HDL-C) levels. Data are presented as mean ± SEM. Statistical analysis was performed using one-way ANOVA. *** *p* < 0.001 compared with the CON group, and ## *p* < 0.01 compared with the HFD group; ns, not significant.

Serum TC and LDL-C levels were significantly higher in the HFD group than in the CON group (*p* < 0.05; Figure 3A,C), confirming the successful induction of hyperlipidemia. No significant differences in serum TC and TG levels were observed between the CP intervention groups and the HFD group (Figure 3A,B). In contrast, serum HDL-C levels were increased in the CP intervention groups, with significantly higher HDL-C levels observed in the HFD+MCP and HFD+HCP groups compared with the HFD group (*p* < 0.05; Figure 3D). This phenomenon may be due to the fact that CP is more likely to modulate hepatic lipid metabolism rather than directly alter circulating lipid levels [22]. Clearly, the changes brought about by lipid metabolism regulation are more enduring.

### 3.3. Serum AST and ALT Levels

**Figure 4:**
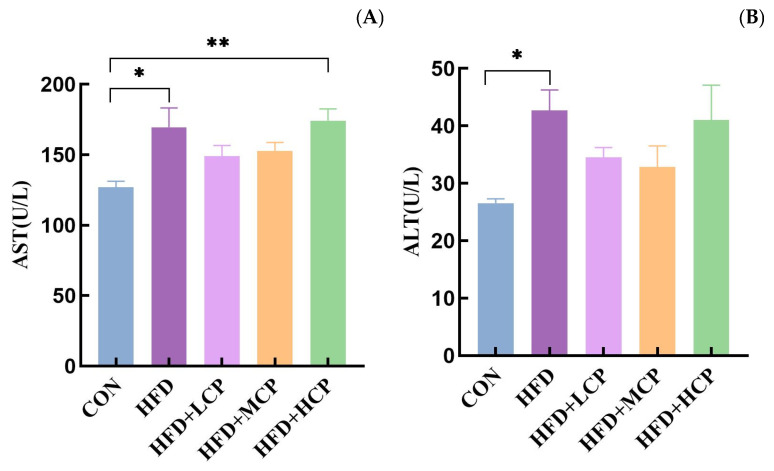
Serum AST and ALT levels in mice. (**A**) Serum aspartate aminotransferase (AST). (**B**) Serum alanine aminotransferase (ALT). Data are presented as mean ± SEM. Statistical analysis was performed using one-way ANOVA. * *p* < 0.05 compared with the CON group. ** *p* < 0.01 compared with the CON group.

When hepatocytes are damaged, ALT and AST are released from the cells into the bloodstream, resulting in elevated serum activities of these aminotransferases [23]. As shown in Figure 4A,B, serum AST and ALT activities in the HFD group were significantly higher than those in the control group (*p* < 0.05), indicating the presence of hepatic injury in the mice. No significant differences in AST and ALT levels were observed between the CP intervention groups and the HFD group.

### 3.4. Serum Uric Acid and Creatinine Levels

**Figure 5:**
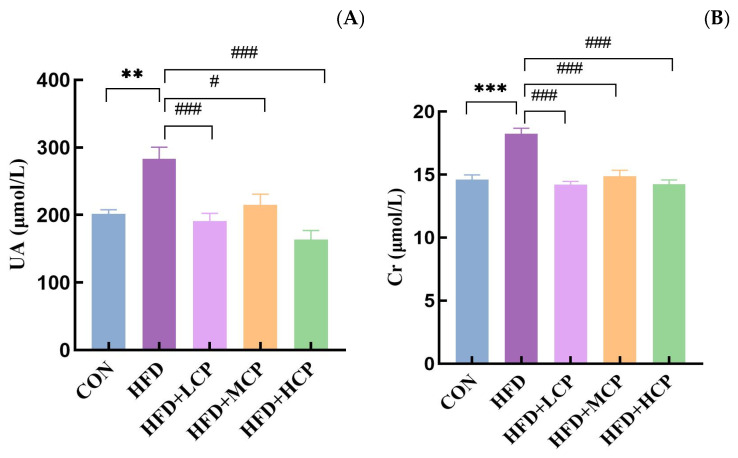
Effects of CP intervention on serum uric acid and creatinine levels. (**A**) Serum uric acid (UA) levels. (**B**) Serum creatinine (Cr) levels. Data are presented as mean ± SEM. Statistical analysis was performed using one-way ANOVA. ** *p* < 0.01 and *** *p* < 0.001 compared with the CON group; # *p* < 0.05 and ### *p* < 0.001 compared with the HFD group.

Serum uric acid and creatinine levels are shown in Figure 5A,B. Compared with the CON group, serum uric acid levels were significantly increased in the HFD group (*p* < 0.05), indicating that the high-fat diet induced hyperuricemia. In contrast, CP supplementation significantly reduced serum uric acid levels compared with the HFD group (*p* < 0.05), with significant decreases observed in the HFD+LCP and HFD+HCP groups (*p* < 0.05; Figure 5A). Similarly, serum creatinine levels were significantly elevated in the HFD group compared with the CON group (*p* < 0.05; Figure 5B), indicating impaired renal function. Compared with the HFD group, CP supplementation significantly reduced serum creatinine levels in all intervention groups (*p* < 0.05).

### 3.5. Effects of CP on Gut Microbial α-Diversity

**Figure 6:**
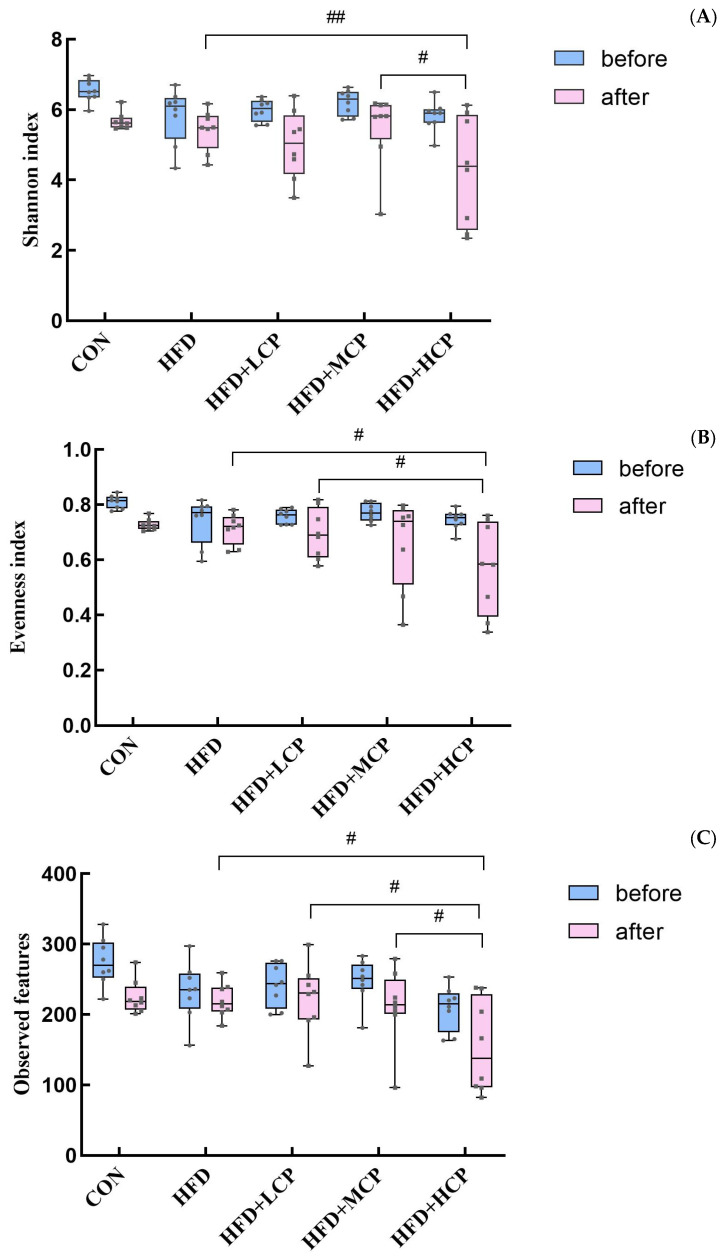
Effects of CP intervention on gut microbial α-diversity before and after intervention. Box plots show the median, interquartile range, and minimum–maximum values. (**A**) Shannon index. (**B**) Evenness index. (**C**) Observed features. Statistical analysis was performed using two-way ANOVA followed by post hoc multiple comparisons. # *p* < 0.05 and ## *p* < 0.01 compared with the HFD group at the same time point.

Alpha diversity analysis showed that there were no significant differences in the Shannon index, Evenness index, or observed features among the groups before the intervention (Figure 6A–C), indicating good comparability in gut microbiota diversity, evenness, and species richness.

After the intervention, compared with the HFD group, significant differences in the alpha diversity of the gut microbiota were observed in the HFD+HCP group. In particular, the Shannon and Evenness indices in the HFD+HCP group showed a significant decrease (*p* < 0.05), while the number of observed features was significantly reduced (*p* < 0.05), suggesting that high-dose CP exerted a significant effect on gut microbiota diversity, evenness, and species richness. These reductions may reflect selective modulation of specific microbial taxa rather than a generalized dysbiotic shift, although the functional implications of these compositional changes remain to be determined [24]. Furthermore, the Shannon index in the HFD+MCP group was significantly higher than that in the HFD+HCP group (*p* < 0.05), indicating that different doses of CP exerted distinct modulatory effects on gut microbiota diversity.

### 3.6. Effects of CP on Gut Microbial β-Diversity

**Figure 7:**
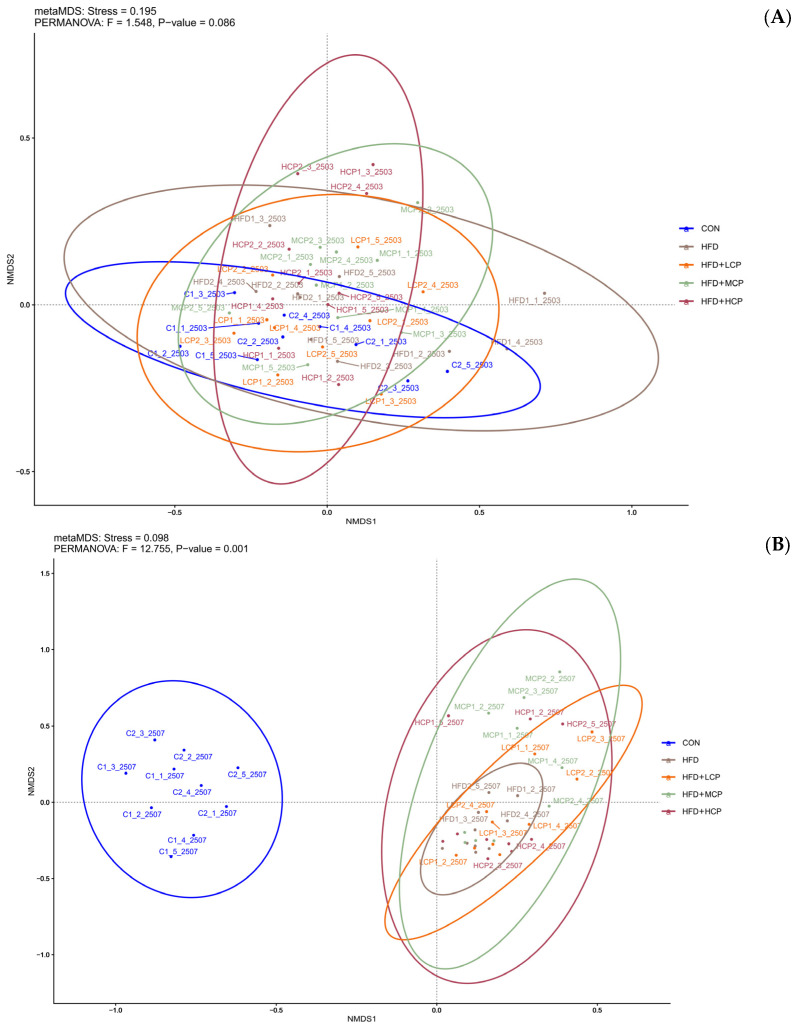
(**A**) Effects of CP intervention on gut microbiota before the intervention. (**B**) Effects of CP intervention on gut microbiota after the intervention. Non-metric multidimensional scaling (NMDS) analysis based on Bray–Curtis dissimilarity was used to assess differences in gut microbial community structure among groups. Statistical significance was evaluated using PERMANOVA.

No obvious separation was observed among groups before the intervention, suggesting comparable baseline gut microbiota composition (Figure 7A). NMDS analysis showed a stress value of 0.098, indicating a reliable ordination (Figure 7B). A tendency toward separation among intervention groups was observed in the two-dimensional space, particularly in the HFD+LCP, HFD+MCP, and HFD+HCP groups, which exhibited a more dispersed distribution, suggesting differences in gut microbial community composition following the interventions. PERMANOVA analysis further confirmed that differences in gut microbiota among groups after intervention were statistically significant (*p* < 0.05), supporting a modulatory effect of CP on gut microbial β-diversity.

### 3.7. Gut Microbiota Composition at the Genus and Phylum Levels

**Figure 8:**
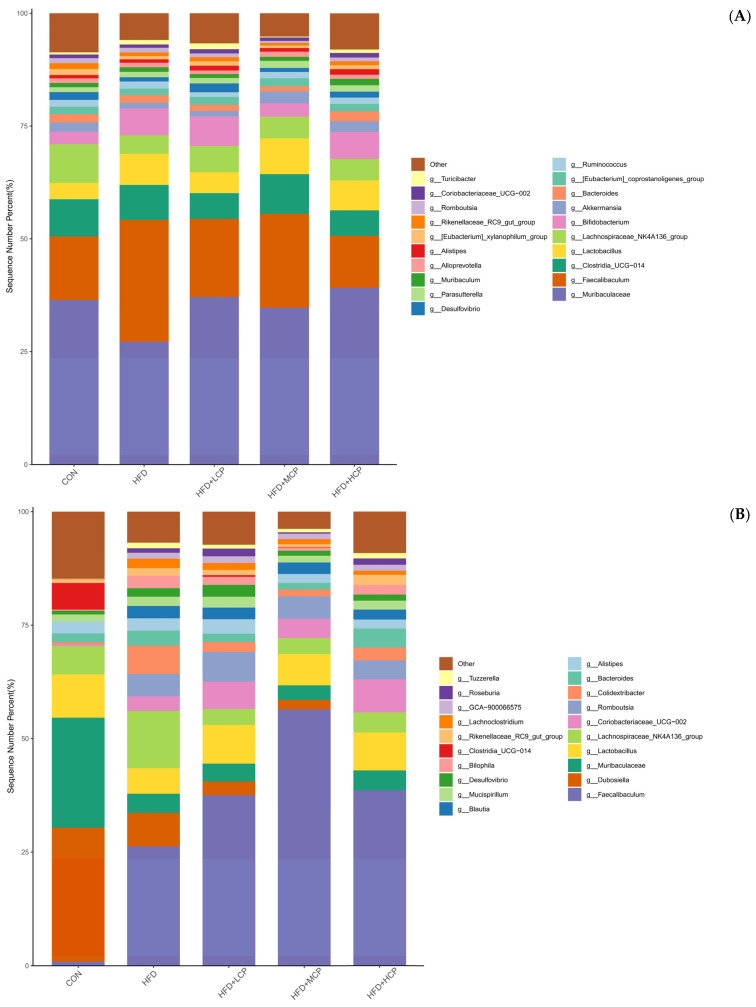

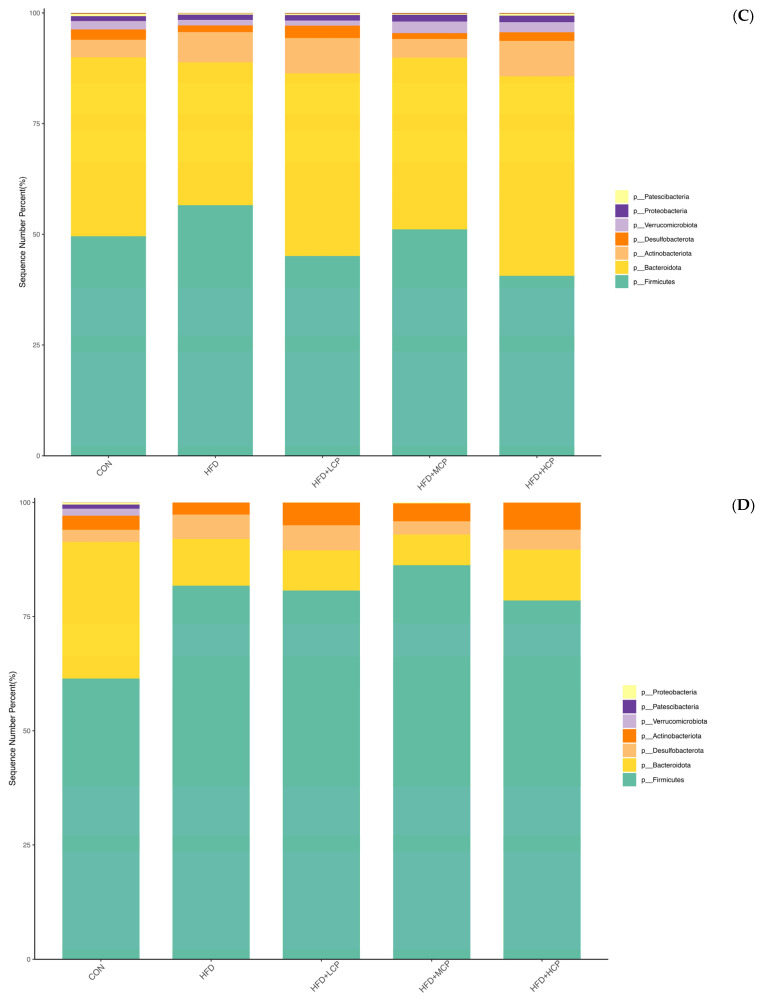

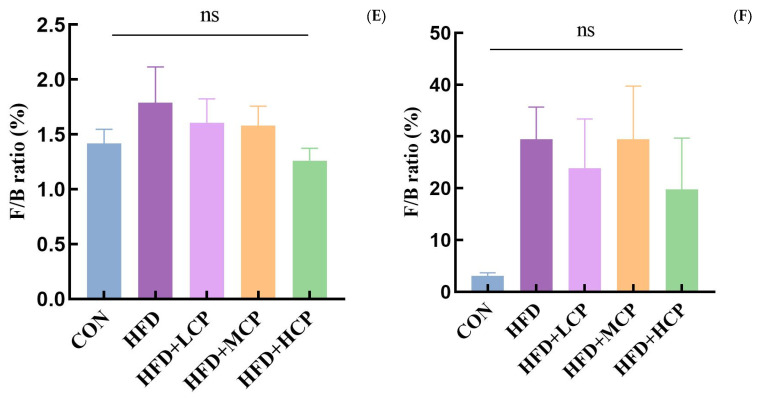
Gut microbiota composition at the genus and phylum levels and the Firmicutes-to-Bacteroidota (F/B) ratio before and after intervention. (**A**,**B**) Relative abundance of gut microbiota at the genus level, expressed as sequence number percent (%). (**C**,**D**) Relative abundance of gut microbiota at the phylum level, expressed as sequence number percent (%). (**E**) Firmicutes-to-Bacteroidota (F/B) ratio before intervention. (**F**) Firmicutes-to-Bacteroidota (F/B) ratio after intervention. Data are presented as mean ± SEM. Statistical analysis for the F/B ratio was performed using one-way ANOVA. ns, not significant.

Analysis of gut microbiota composition at the genus level showed that (Figure 8A), before the intervention, the overall microbial composition was similar among groups, indicating good baseline comparability. Following CP supplementation, several dominant genera, including *Faecalibaculum*, *Muribaculaceae*, *Dubosiella*, and *Lactobacillus*, were consistently detected across all groups (Figure 8B). Compared with the CON group, the HFD group exhibited a reduced relative abundance of *Muribaculaceae* and an increased abundance of *Faecalibaculum*, consistent with microbial alterations commonly associated with high-fat diet intake. In the CP intervention groups, a higher relative abundance of *Lactobacillus* was observed compared with the HFD group, suggesting a potential shift in microbial composition. Notably, *Faecalibaculum* abundance was highest in the MCP group. At the phylum level, *Firmicutes* and *Bacteroidota* predominated across all experimental groups (Figure 8C,D). Compared with the CON group, the relative abundance of *Firmicutes* was increased in the HFD group, while *Bacteroidota* decreased accordingly, showing typical characteristics of a microbiota associated with a high-fat diet. In the CP intervention groups (HFD+LCP, HFD+MCP, and HFD+HCP), *Firmicutes* remained the predominant phylum. Compared with the HFD group, only slight variations in the relative abundances of *Firmicutes* and *Bacteroidota* were observed among the intervention groups, suggesting that CP had a limited influence on gut microbiota composition at the phylum level.

At baseline, no significant differences in the Firmicutes-to-Bacteroidota (F/B) ratio were observed among the groups (Figure 8E). After the intervention, the CP intervention groups (HFD+LCP, HFD+MCP, and HFD+HCP) exhibited a decreasing trend in the F/B ratio compared with the HFD group, although the differences were not statistically significant (Figure 8F).

### 3.8. Correlations Between Gut Microbiota and Metabolic Parameters

**Figure 9:**
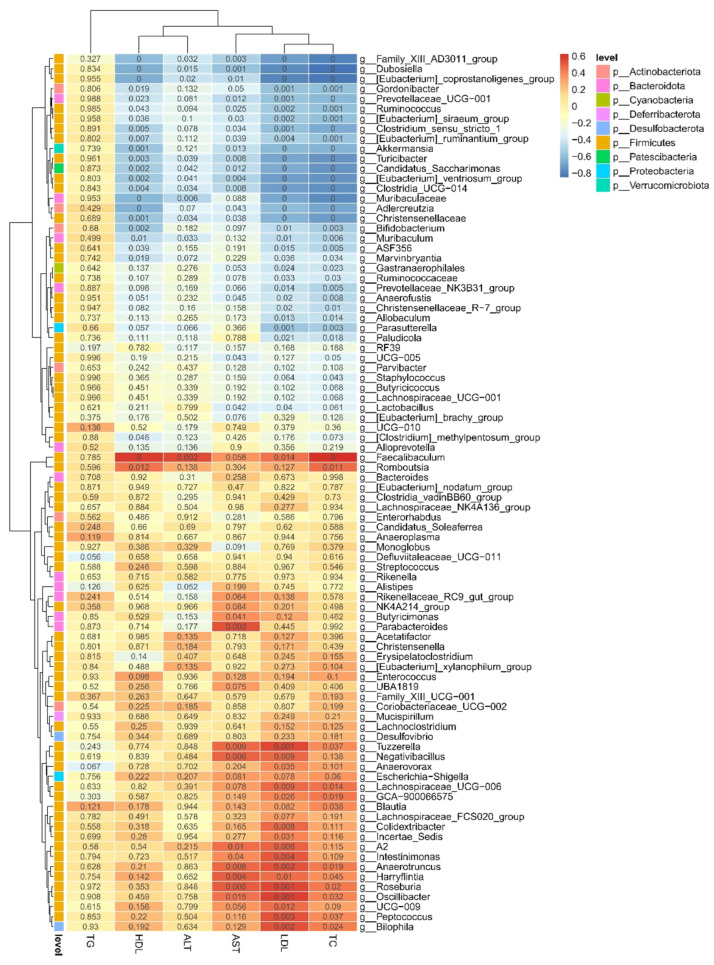
Correlations between gut microbiota and metabolic parameters. Pearson correlation analysis was performed to evaluate the associations between the relative abundance of gut microbial genera and metabolic parameters. Correlation coefficients are displayed as a heatmap, with red indicating positive correlations and blue indicating negative correlations.

To further examine the relationships between gut microbiota at the genus level and serum biochemical indicators, correlation analysis was performed, as shown in Figure 9. The results showed that the relative abundance of *Akkermansia* was negatively correlated with serum TC, LDL-C, and AST levels (*p* < 0.05). In addition, *Bifidobacterium* exhibited significant negative correlations with serum TC and LDL-C levels (*p* < 0.05). A significant negative correlation was also observed between the relative abundance of *Lactobacillus* and serum LDL-C and AST levels (*p* < 0.05). These findings indicate potential associations between specific bacterial genera and metabolic parameters; however, these correlations do not imply causation and should be interpreted with caution.

## 4. Discussion

This study evaluated the effects of cricket powder (CP) supplementation on blood lipid parameters and gut microbiota structure in mice with high-fat diet-induced hyperlipidemia. The results demonstrated that, compared with the HFD group, CP supplementation was associated with non-significant downward trends in serum total cholesterol (TC), triglycerides (TG), low-density lipoprotein cholesterol (LDL-C), aspartate aminotransferase (AST), and alanine aminotransferase (ALT) levels. In contrast, there were significant changes in the gut microbiota at the genus level, suggesting a potential association between CP supplementation and gut microbiota modulation, which may be related to changes in lipid metabolism, although the differences were not statistically significant.

CP is rich in chitin, a structural polysaccharide that is poorly digested and absorbed by the host [25]. Owing to its physicochemical properties resembling those of dietary fiber, chitin has been suggested to potentially influence host lipid metabolism through gut microbiota-mediated mechanisms [26]. In this study, although no significant differences in *Bifidobacterium* abundance were observed at the genus level, correlation analysis revealed a significant negative correlation between *Bifidobacterium* abundance and TC and LDL-C. Similarly, the relative abundance of *Lactobacillus* was negatively correlated with LDL-C and AST levels. These findings suggest that these genera may be associated with metabolic processes rather than directly exerting metabolic effects through marked changes in relative abundance. Previous studies have shown that *Lactobacilli* and *Bifidobacteria* species can ferment complex polysaccharides and produce short-chain fatty acids (SCFAs), such as acetic acid and propionic acid [27]. SCFAs have been reported to be involved in lipid metabolism, including the regulation of hepatic lipid synthesis and cholesterol metabolism [28]. Therefore, the negative correlations observed between specific gut microbial genera and serum lipid parameters in this study may be partly related to components of CP, such as chitin, which has been suggested to act as a fermentable substrate and potentially promote short-chain fatty acid (SCFA) production. However, the precise mechanisms underlying these associations remain to be elucidated. In addition, SCFA levels were not measured in the present study; therefore, the proposed SCFA-related mechanisms remain speculative.

Furthermore, correlation analysis revealed significant negative associations between *Akkermansia* abundance and serum TC, LDL-C, and AST levels. *Akkermansia* is widely recognized as a beneficial gut bacterium that is closely linked to intestinal barrier integrity and overall metabolic health [29]. Previous studies have suggested that *Akkermansia* may contribute to the alleviation of chronic low-grade inflammation associated with metabolic diseases by degrading mucins in the intestinal mucus layer, promoting epithelial cell renewal, and enhancing intestinal barrier function [30]. Previous studies have demonstrated that increased *Akkermansia* abundance is associated with improvements in lipid profiles, protection against liver injury, and reduced inflammatory responses [31]. In this context, the findings of the present study suggest that CP intake may be associated with changes in lipid metabolism and liver-related parameters, potentially in relation to *Akkermansia*-associated gut microbiota alterations.

In addition to its regulatory effects on the gut microbiota, the lipid-lowering effects of CP may be associated with its antioxidant properties. Oxidative stress is widely recognized as an important factor driving the pathological progression of atherosclerosis [32]. A high-fat diet can lead to excessive accumulation of lipids and increase the production of reactive oxygen species, thereby inducing lipid peroxidation reactions. Oxidation modification of LDL-C can further impair endothelial function and promote foam cell formation [33]. Accordingly, dietary interventions with antioxidant properties may help alleviate lipid oxidative damage. Previous studies have demonstrated that cricket protein powder exhibits notable antioxidant activity and can effectively inhibit lipid peroxidation [34]. In addition, cricket protein has been shown to release bioactive peptides during gastrointestinal digestion or enzymatic hydrolysis, which exhibit antioxidant and antihypertensive activities and may be involved in lipid metabolism-related processes [35]. However, antioxidant-related parameters were not measured in the present study; therefore, the proposed antioxidant mechanism should be considered a hypothesis and requires further experimental validation. Research has indicated that peptides derived from crickets may be involved in the regulation of cholesterol synthesis by modulating HMG-CoA reductase activity [36]. Overall, these findings suggest that CP intake may be associated with lipid metabolism-related changes, possibly in relation to gut microbiota modulation and antioxidant-related processes. However, these proposed mechanisms are speculative and not directly supported by the present data, and further studies are required to elucidate the underlying biological mechanisms.

## 5. Conclusions

The present study demonstrated that gavage administration of CP was associated with alterations in the intestinal microbiota of mice with hyperlipidemia induced by a high-fat diet. Although the serum lipid indicators showed only a downward trend and did not reach statistical significance, the α diversity of the intestinal microbiota significantly changed after the intervention, and the β diversity also showed a significant separation, indicating that CP may influence the structure of the intestinal microbial community. Correlation analysis further revealed that beneficial bacterial genera such as *Lactobacillus*, *Bifidobacterium*, and *Akkermansia* had a negative correlation with lipid-related parameters. These results suggest that CP may influence lipid-related metabolic parameters primarily through modulation of gut microbiota composition; however, no statistically significant changes were observed in serum lipid levels. The relatively small sample size (*n* = 6 per group) may limit the statistical power of this study and should be considered when interpreting the results. Future studies are needed to further elucidate the underlying mechanisms linking CP, gut microbiota, and metabolic regulation, as well as to identify the key bioactive components responsible for these effects.

## Data Availability

The original contributions presented in this study are included in this article. Further inquiries can be directed to the corresponding author.
